# Hypercalcemic Crisis Due to a Giant Intrathyroidal Parathyroid Adenoma, with Postsurgical Severe Hypocalcemia and Hungry Bone Syndrome: A Case Report

**DOI:** 10.3390/clinpract14010015

**Published:** 2024-01-22

**Authors:** Vasileios Papanikos, Elli Papadodima, Dimitra Bantouna, Rodis D. Paparodis, Sarantis Livadas, Nicholaos Angelopoulos, Evangelos Karvounis

**Affiliations:** 1 Department of Otorhinolaryngology, School of Medicine, General University Hospital of Patras, 26504 Patras, Greece; 2 Division of Endocrinology, Diabetes and Metabolism, Euroclinic Hospital, 11521 Athens, Greece; 3 Endocrinology, Diabetes and Metabolism Clinics, Private Practice, 26221 Patras, Greece; 4 Center for Diabetes and Endocrine Research, College of Medicine and Life Sciences, University of Toledo, Toledo, OH 43614, USA; 5 Endocrinology, Diabetes and Metabolism Clinics, Private Practice, 11524 Athens, Greece; 6 Endocrinology, Diabetes and Metabolism Clinics, Private Practice, 65302 Kavala, Greece; 7 Center of Excellence in Endocrine Surgery, Euroclinic Hospital, 11521 Athens, Greece; karvounis@endocrinesurgeon.gr

**Keywords:** giant parathyroid adenoma, hypercalcemic crisis, hungry bone syndrome

## Abstract

Background: Parathyroid adenoma is the most common cause of hypercalcemia and rarely leads to a hypercalcemic crisis, which is an unusual endocrine emergency that requires timely surgical excision. Case presentation: A 67-year-old male was admitted to the ER of the Euroclinic Hospital, Athens, Greece, because of elevated calcium levels and a palpable right-sided neck mass, which were accompanied by symptoms of nausea, drowsiness, and weakness for six months that increased prior to our evaluation. A gradual creatinine elevation and decreasing mental state were observed as well. The initial laboratory investigation identified severely elevated serum calcium (3.6 mmol/L) levels consistent with a hypercalcemic crisis (HC) and parathyroid hormone PTH (47.6 pmol/L) due to primary hyperparathyroidism. Neck ultrasonography (USG) identified a large, well-shaped cystic mass in the right thyroid lobe. With a serum calcium concentration of 19.5 mg/dL and a PTH of 225.3 pmol/L, the patient underwent partial parathyroidectomy and total thyroidectomy, which decreased serum calcium and PTH to 2.5 mmol/L and 1.93 pmol/L, respectively. Histology revealed a giant intrathyroidal cystic parathyroid adenoma, which was responsible for the hypercalcemic crisis. Postoperatively, the patient developed severe biochemical and clinical hypocalcemia, with calcium concentrations as low as 1.65 mmol/L, consistent with hungry bone syndrome (HBS), which was treated with high doses of intravenous calcium gluconate and oral alfacalcidol, and a slow recovery of serum calcium. After discharge, parathyroid function recovered, and symptomatology resolved entirely in more than one month. Discussion/conclusions: We present a case involving an exceptionally large intrathyroidal parathyroid adenoma that is characterized by clinical manifestations that mimic malignancy. The identification and treatment of such tumors is challenging and requires careful preoperative evaluation and postoperative care for the risk of hungry bone syndrome.

## 1. Introduction

Parathyroid adenomas are the most common cause of primary hyperparathyroidism (PhP), a relatively common endocrine disorder causing hypercalcemia of varying degrees [1]. PhP is associated with cardiac, renal, and skeletal complications, while peptic ulcer disease is common in this population [1]. Rarely, hypercalcemia may lead to a hypercalcemic crisis, a life-threatening emergency that requires immediate medical attention [2]. Typical laboratory findings of PhP include elevated serum calcium and parathyroid hormone (PTH). The surgical resection of parathyroid adenomas, especially in specialized parathyroid surgery centers, results in the prompt resolution of hypercalcemia in 95% of cases, but in order to achieve this, the correct preoperative localization of this tumor is necessary [3,4]. The standard preoperative imaging modalities employed in that regard include a neck ultrasound (USG), followed by parathyroid scintigraphy (Tc-Sestamibi) [4]. Occasionally, parathyroidectomy leads to postoperative hypocalcemia, an electrolytic abnormality that could be detrimental; hence, its timely recognition and treatment are of utmost importance [5]. Hungry bone syndrome (HBS) is defined as prolonged and severe hypocalcemia after parathyroidectomy and requires the presence of a total serum calcium of less than 2.1 mmol/L for more than four days postoperatively [6].

## 2. Case Presentation 

A 67-year-old Caucasian male attended our outpatient endocrine clinics for the evaluation of severely elevated serum calcium levels, along with a palpable right-sided neck mass. His main symptoms consisted of nausea and generalized weakness of increasing intensity for the past six months. His past medical history consisted of hypertension, hyperlipidemia, and hyperuricemia, while his medical treatment included a combination of Olmesartan with 20/12.5 mg of hydrochlorothiazide daily, 10 mg of rosuvastatin daily and 80 mg of febuxostat daily. Lately, the patient started using 10 mg of rabeprazole daily over the counter for the alleviation of heartburn. Further history revealed two episodes of nephrolithiasis in the past. The first episode (twenty years ago) was treated surgically by removing the stones from the right kidney while the second (three years ago) was treated with lithotripsy. Physical examination revealed firm, painless, and mobile swelling in the right thyroid lobe without palpable lymphadenopathy. 

### 2.1. Diagnostic Assessment

**Figure 1:**
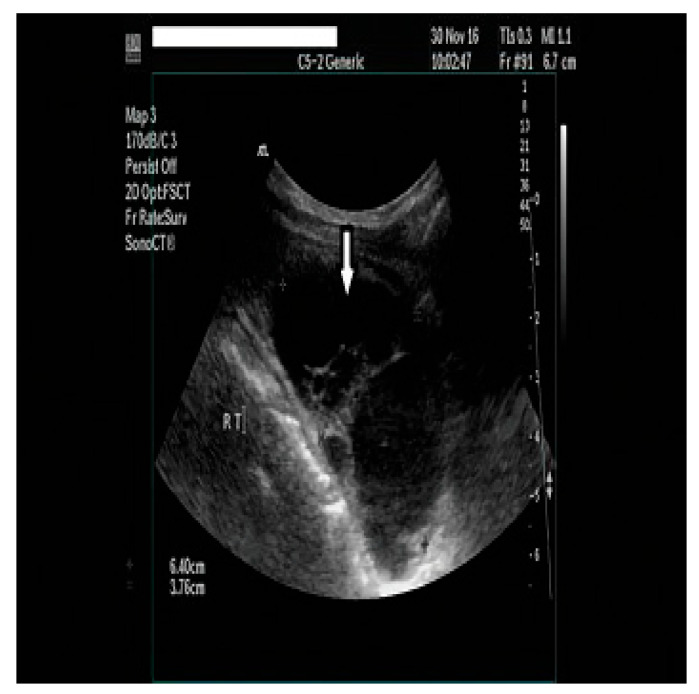
A neck ultrasonography (USG) showed a 6.90 × 3.05 × 2.60 cm multilobulated cyst in the lower part of the right thyroid lobe.

**Figure 2:**
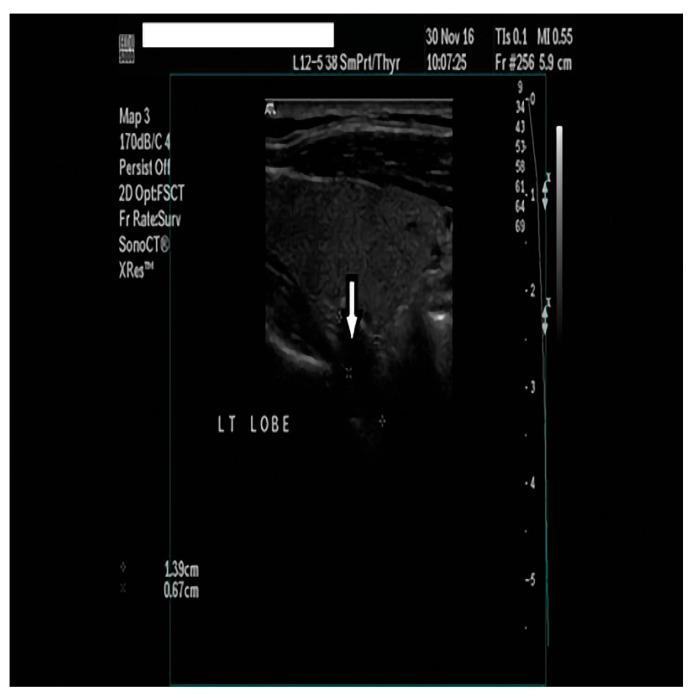
A neck ultrasound revealed a 1.4 × 0.7 × 0.5 cm oval-shaped, hypoechoic mass below the left thyroid lobe.

**Figure 3:**
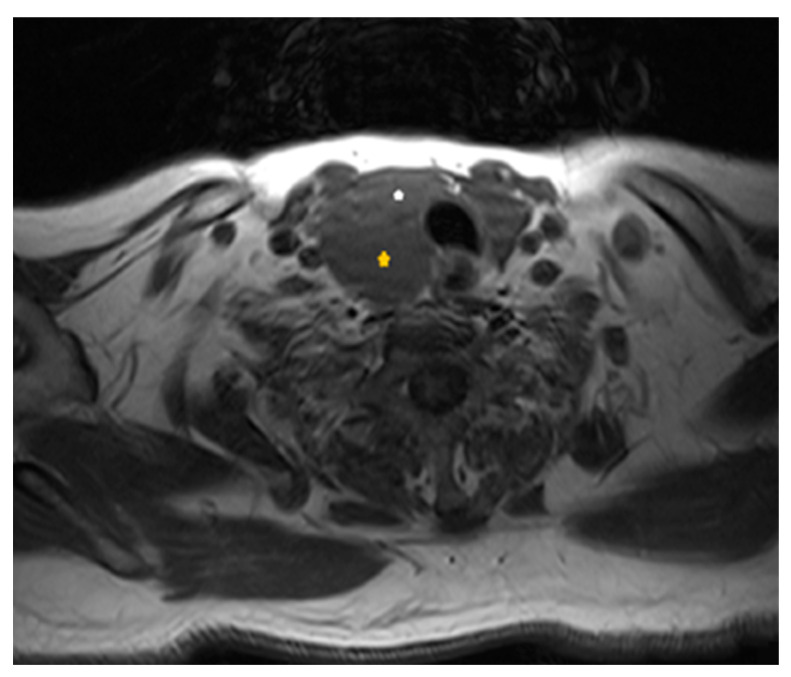
Axial T1-weighted scan at the level of the thyroid gland: yellow asterisk shows the extrathyroidal large parathyroid cystic mass, posteriorly to the right side of the thyroid gland (white asterisk).

Our initial laboratory examination was within the overall reference range, with the pronounced exception of serum calcium and parathyroid hormone concentrations, which were markedly elevated at 3.6 mmol/L (Ref. 2.1–2.5) and 47.6 pmol/L (Ref. 1.0–6.5), respectively (please see Table 1 for the baseline chemistries). In addition, urine was collected over 24 h to assess for the presence of concurrent pheochromocytoma; total urinary metanephrine concentrations were slightly elevated at 2576 nmol/24 h (Ref. 449–2264). The measurement was performed using a radioimmunoassay. Subsequently, the patient underwent neck ultrasonography (USG), which showed a 6.90 × 3.05 × 2.60 cm multilobulated, well-defined cystic mass in the lower half of the right thyroid lobe without features that were suspicious for malignancy (microcalcifications, irregular shape, abnormal lymph nodes, or increased intralesional vascularity) (Figure 1). In addition, the thyroid gland was found to be enlarged and multinodular, without nodules that were suspicious for malignancies either. An ultrasound evaluation of the kidneys and adrenals was performed without abnormal findings, while a computed tomography scan (CT) revealed a 13.5 mm mass in the right adrenal gland. Because the Euroclinic General Hospital is a private clinic, the patient could not undergo a 99-m-Tc-methoxy-isobutyl-isonitrile (MIBI) scintigraphy for financial reasons. The patient was aggressively hydrated and treated with cinacalcet tablets (60 mg/day), and he was discharged after 3 days with the same dosage of oral cinacalcet, a calcium level of 3.1 mmol/L, and instructions to follow up in a week in order to prepare for parathyroid surgery at our Center of Excellence in Endocrine Surgery. Unfortunately, the patient returned to the hospital 10 days later with an altered mental status, elevated serum calcium levels of 4.9 mmol/L, and severe deterioration of his renal function (serum creatinine 495 Umol/L). A second neck ultrasound and neck magnetic resonance imaging (MRI) were performed, and this additionally disclosed a previously unknown 1.4 × 0.7 × 0.5 cm oval-shaped mass located under the left lower pole of the thyroid gland (Figure 2 and Figure 3). The patient was taken to the operating room for parathyroidectomy. Initially, the surgeon removed only the above mass, but the intraoperative PTH concentration failed to drop significantly (225.3 to 135.4 pmol/L). Given the presence of a potential intrathyroidal adenoma and the multinodular nature of his thyroid gland, a decision was made to extend the surgery to a total thyroidectomy. This resulted in a curative drop in the intraoperative parathyroid hormone concentration to 22.4 pmol/L. 

### 2.2. Treatment 

During the first admittance at our emergency department, the patient received hydration with a saline infusion, along with intravenous furosemide (80 mg/day) and cinacalcet tablets (60 mg/day). Calcium concentrations dropped to 3.2 mmol/L within 24 h. After 3 days, the serum calcium concentration decreased further to 3.1 mmol/L, and the patient was discharged on the same regimen. Due to personal reasons, the patient did not opt to undergo immediate surgery, which was against our medical advice, and he left on cinacalcet (60 mg daily) while being advised to monitor serum calcium concentrations weekly. Ten days after discharge, the patient developed gastroenteritis and discontinued his treatment for 3 days; he returned to the hospital for a second time and was normotensive but unconscious (Glasgow Coma Scale = 4), with a serum calcium concentration of 4.9 mmol/L. Aggressive intravenous hydration was resumed, along with subcutaneous calcitonin (100 IU three times daily), intravenous methylprednisolone (40 mg twice daily), and intravenous zoledronic acid (4 mg once). Serum calcium concentrations dropped to 4.3 mmol/L after 48 h, while serum creatinine concentrations remained unchanged (495 Umol/L). Subsequently, his family was informed about the severity of the situation, and written consent was obtained to perform an emergency surgical neck exploration carried out by an endocrine surgeon. Before the induction of anesthesia, calcium and parathyroid hormone concentrations were 3.5 mmol/L and 225.3 pmol/L, respectively. Initially, the surgeon only removed the left 1.4 cm oval mass, but the intraoperative PTH concentration failed to drop significantly (135.4 pmol/L). Consequently, the surgeon extended the operation to a total thyroidectomy (due to the multinodular shape of the thyroid gland and a potential intrathyroidal parathyroid adenoma), producing a curative drop in parathyroid hormone concentrations according to the Miami criterion (22.4 pmol/L, 50% decline) [3,5,7].

### 2.3. Outcome and Follow-Up 

**Figure 4:**
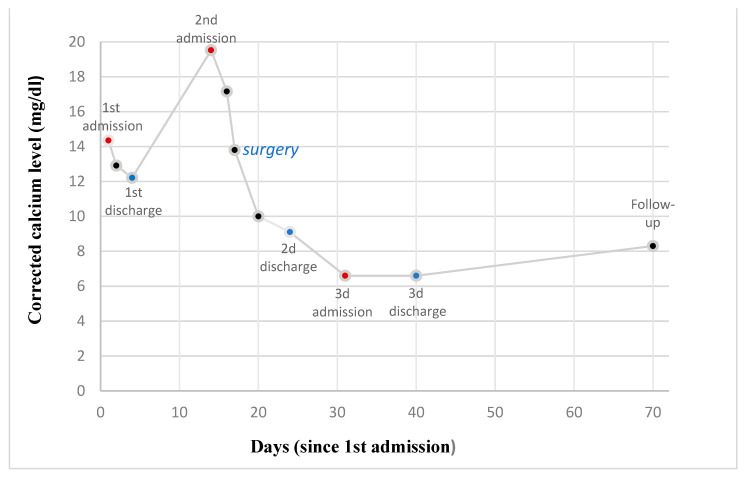
Variations in the concentration of corrected calcium levels (mg/dL) as a function of time (days) since 1st admission.

Postoperatively, the patient’s symptoms improved and cinacalcet was discontinued, while albumin-corrected calcium (2.5 mmol/L) and PTH (1.93 pmol/L) concentrations reached normal values within two days. After six days of postoperative care, the patient was discharged home with a serum calcium concentration of 2.3 mmol/L and was put on treatment with oral calcium gluconate/calcium carbonate (1 g, 3 times daily), alfacalcidol (1 mcg, 3 times daily), cholecalciferol (25,000 IU/amp once weekly), and levothyroxine (125 mcg daily). However, one week later, he returned to the emergency department because of severe numbness, cramps, and diffuse paresthesia, and he was found to exhibit both Chvostek’s and Trousseau’s signs. The corrected serum calcium level was 1.6 mmol/L, PTH was 2.67 pmol/L, phosphorus was 1.5 mmol/L (Ref. 0.8–1.4 mmol/L), creatinine was 274 Umol/L, TSH was 19.3 mlU/mL, and magnesium was 0.5 mmol/L. We administered high doses of intravenous calcium gluconate (1700 mg daily) and oral alfacalcidol (3 mcg daily) without substantial improvements in calcium concentrations, even though the clinical manifestations were corrected. After a nine-day inpatient stay, he left the hospital against medical advice with a serum calcium concentration of 1.6 mmol/L. The discharge regimen consisted of oral calcium citrate, malate and carbonate, cholecalciferol, alfacalcidol, thyroxine (125 mcg daily), and magnesium supplements (magnesium citrate 200 mg twice daily), and this regimen was meant to be subject to outpatient corrections according to daily laboratory measurements. Calcium concentrations increased gradually and reached the reference within two months postoperatively, while TSH was 1.3 mIU/L at the same time. The last neck ultrasound revealed no thyroid remnants or further enlarged parathyroid glands, while new CT and MRI scans did not identify any abnormalities in the adrenal glands. Postoperative 24 h urine total metanephrine levels were normal; thus, the slightly increased concentration of baseline urinary metanephrines was attributed to the excessive systemic stress of the patient. In order to evaluate the possibility of the presence of MEN2 syndrome, RET proto-oncogene sequencing was performed and did not reveal the presence of any mutations. Six years postoperatively, the patient remains asymptomatic, with the latest serum calcium (2.4 mmol/L) and PTH (5.4 pmol/L) concentrations within reference ranges and without symptoms nor complications (Figure 4).

### 2.4. Histology

**Figure 5:**
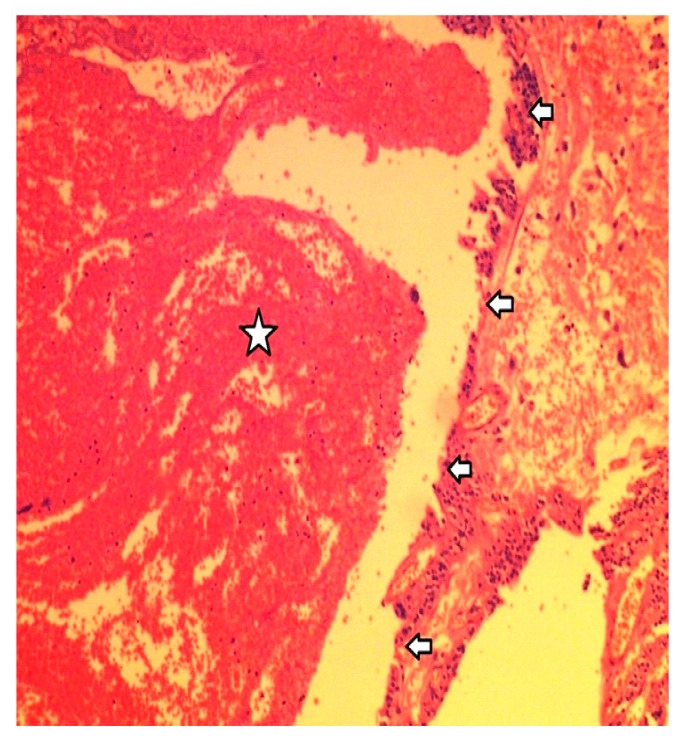
From the histological examination, the cystic hemorrhagic degeneration of the adenoma was identified. Arrows mark the cyst’s wall, while the star denotes hemorrhagic cystic contents.

**Figure 6:**
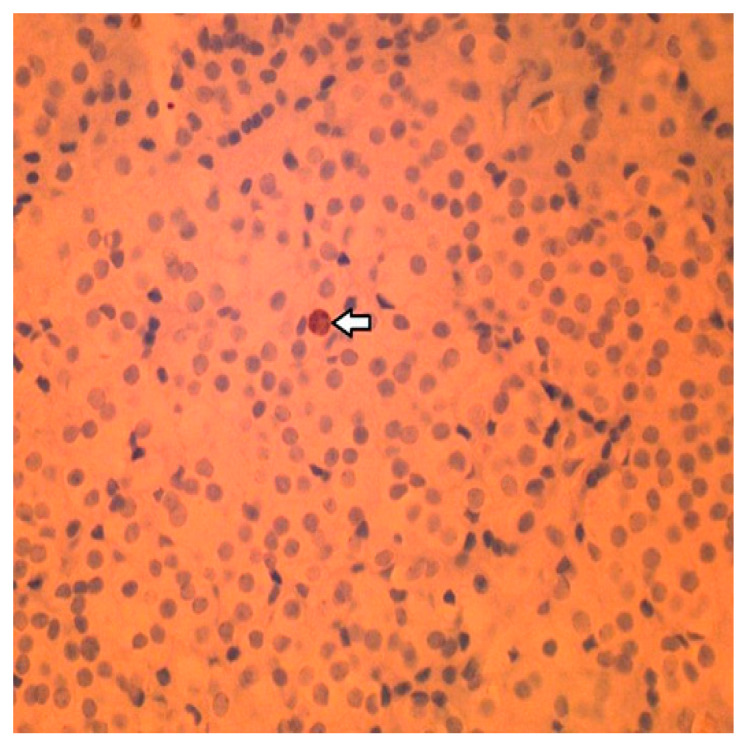
Immunohistochemistry staining: Less than 1% of the cells stained positive for the cell proliferation marker Ki-67. Arrow indicates the Ki-67-positive cell in this field.

The histopathologic report revealed a mass adjacent to the thyroid gland, measuring 5.5 × 5.0 × 2.0 cm, weighing 20 g, contained in a thin hemorrhagic capsule, and without necrotic features (Figure 5). The mass comprised hyperplastic chief cells and clear or acidophilic cells, and these were in trabecular, lobular, follicular, and alveolar formations with nuclear polymorphisms. Based on the size of the tumor and the presence of a single cell type and cystic hemorrhages, a diagnosis of parathyroid carcinoma was entertained. The tumor stained positive for cyclin D1 in many cells but did so rarely for p16, whereas Ki-67 was detected in 0–1% of cells (Figure 6). The final pathological diagnosis was that of a giant intrathyroidal parathyroid adenoma of uncertain biological behavior, with cystic degeneration. In addition, the left oval-shaped mass under the left lower pole of the thyroid gland was also proven to be a parathyroid adenoma. The pathological evaluation of the thyroid gland revealed the presence of multinodular goiter, without features of malignancy in the nodules.

## 3. Discussion

Primary hyperparathyroidism is an uncommon endocrine disorder caused by the increased and unregulated production of parathyroid hormone; its most common cause is the presence of a parathyroid adenoma in approximately 80% of cases, while parathyroid carcinoma may also be found on rare occasions [8]. Its most common feature is the presence of hypercalcemia, which is responsible for gastrointestinal symptoms (constipation, abdominal cramps, pain, and peptic ulcer disease); nephrolithiasis; and osteoporosis with fractures, and when severe, it could lead to pronounced hypercalciuria and dehydration, mental fog, and altered mental status [1,9]. A hypercalcemic crisis is a rare endocrine emergency, and it is defined as the presence of an excessively increasing albumin-corrected serum calcium concentration (above 46 g/L) with concomitant multiorgan dysfunction (pancreatitis, gastrointestinal, renal, neurologic, or cardiovascular sequelae) [1,2,10]. Our patient exhibited renal and neurological dysfunctions, expressed as markedly elevated creatinine levels and impaired consciousness. The timely diagnosis and treatment of this urgent condition are very important in order to prevent dangerous and potentially fatal consequences [2]. The treatment of hypercalcemic crises includes aggressive hydration with isotonic fluids accompanied by intravenous bisphosphonates or calcitonin and calciuretics, such as furosemide. Long-term control of the disease requires the surgical resection of the responsible tumor, which is often curative when parathyroid cancer is not involved. 

A major difficulty in the surgical management of PhP is the correct preoperative localization of the tumor, which produces excess parathyroid hormones. Preoperative neck ultrasound (USG) and 99mTc-MIBI may be helpful in this regard, even though their accuracy is far from perfect [4]. Clinical improvements rapidly occur after surgical treatment, especially in symptomatic patients and those with complications related to hyperparathyroidism, such as kidney stones, osteoporosis, or significant hypercalcemia (>2.9 mmol/L) [9]. Asymptomatic hyperparathyroidism should be operated on in patients younger than 50 years, those with creatinine clearances of < 60 mL/min, and those with serum calcium levels of > 0.25 mmol/L, above the highest end of the reference range, while some experts advocate the use of surgery in patients with pronounced hypercalciuria (24 h urinary calcium > 1530 nmol/24 h) as well [1,4,9]. On the other hand, patients who do not wish to undergo surgical treatment and those who are not appropriate candidates for surgery (comorbidities and milder disease) could be followed up clinically and receive medical therapy alone. This consists of long-term treatment with antiresorptives (bisphosphonates and denosumab) to prevent fractures, along with calcimimetics (cinacalcet), if needed, to maintain a safe serum calcium concentration level [4,10]. Similar strategies are also used in patients with mild hypercalcemia and those without complications from hyperparathyroidism. However, surgical intervention is the only recommended definitive treatment for primary hyperparathyroidism with manifestations [9,11,12]. Our case involves an uncommon, functional, large, cystic parathyroid mass, which accounts for 1–5% of neck masses. These are predominantly found in women, with 61.4% being non-functioning, and they may reach a diameter of up to 15 cm [13]. A strong clinical suspicion of parathyroid carcinoma was raised in the present case because of the extremely unusual, giant size of the lesion (5.5 × 5.0 × 2.0 cm and 20 g weight), the pronounced hypercalcemia, and extremely high PTH concentrations. Luckily, the tumor was proven to be an atypical cystic parathyroid adenoma exhibiting uncertain behavior, which did not show any evidence of malignant behavior over a period of 6 years during follow-up. In order to obtain a therapeutic drop in serum PTH, we had to extend our operation to include a total thyroidectomy, because the original surgery was inadequate for correcting the hypercalcemia. 

Moreover, we ought to emphasize the importance of measuring PTH concentrations before, during, and after the operation in order to ensure the appropriate decrease. After the surgical correction of his hyperparathyroidism, our patient presented with severe and prolonged hypocalcemia, a postoperative complication known as hungry bone syndrome (HBS), which occurs relatively rarely [14]. While there is no clear definition for this rare entity, HBS is considered in patients whose serum calcium level is below 2.1 mmol/L for more than four days after thyroidectomy or parathyroidectomy [6,12]. Risk factors for HBS are a patient age of > 60 years; a preoperative serum PTH level of > 100 pmol/L; a preoperative serum alkaline phosphatase level three times higher than the upper reference limit; and/or radiological findings of severe bone involvement, such as fractures, bone lysis, or brown tumors [6]. In such cases, hypocalcemia should be treated urgently with high doses of calcium supplements and oral vitamin D analogs, but if serum calcium levels fall below 1.9 mmol/L or if symptoms or associated electrocardiographic changes occur, intravenous calcium (chloride or gluconate) should be administered as well. Finally, we should keep in mind that following any thyroidectomy or parathyroidectomy, the immediate postoperative period is crucial for the early detection and treatment of any degree of hypocalcemia, including the following rare but most dangerous form: hungry bone syndrome. 

## 4. Conclusions

Parathyroid adenoma is the most common cause of hypercalcemia and can rarely lead to a hypercalcemic crisis—an unusual endocrine emergency that requires immediate surgical management. Regular serum measurements of parathyroid hormones, calcium, and albumin are necessary in order to avoid detrimental consequences. Hungry bone syndrome is a rare complication (a prolonged state of hypocalcemia) following thyroidectomy or parathyroidectomy and needs to be screened, with postoperative serum calcium measurements carried out in all patients. This report highlights the rare presence of a giant intrathyroidal parathyroid adenoma, for which its clinical picture resembled cancer and its resection led to hungry bone syndrome.

## Figures and Tables

**Table 1:** Laboratory evaluation.

Test Name	Initial Laboratory Values	Highest Values Observed	Reference Range
Calcium, serum (Ca)	3.6	4.9	2.1–2.5 mmol/L
Parathyroid hormone (PTH)	47.6	225.3	1.0–6.5 pmol/L
Creatinine, serum (CREA)	97	495	53–124 Umol/L
Alkaline phosphatase (ALP)	85	86	23–129 U/L
Alboumin (ALB)	44	46	35–50 g/L
Magnesium (MG)	0.7	0.5	0.6–1.0 mmol/L
Phosphorus (PHOS)	1.2	1.5	0.8–1.3 mmol/L
Thyroid-stimulating hormone (TSH)	N/A	19.3	0.3–4.7 mlU/L
24 h urine total metanephrines	2576	2576	449–2264 nmol/24 h

## Data Availability

Original data generated and analyzed during this study are included in this published article. All data are available upon request.
